# The Role of Plain Radiography in Assessing Aborted Foetal Musculoskeletal Anomalies in Everyday Practice

**DOI:** 10.3390/jimaging10100242

**Published:** 2024-09-27

**Authors:** Benedetta Rossini, Aldo Carnevale, Gian Carlo Parenti, Silvia Zago, Guendalina Sigolo, Francesco Feletti

**Affiliations:** 1 Unit of Radiology, S. Maria delle Croci Hospital, Ausl Romagna, 48121 Ravenna, Italy; benedetta.rossini@auslromagna.it (B.R.); giancarlo.parenti@auslromagna.it (G.C.P.); 2 Dipartimento di Medicina Traslazionale e per la Romagna, Università degli Studi di Ferrara, 44121 Ferrara, Italy; crnlda@unife.it (A.C.); sglgdl@unife.it (G.S.); 3 Unit of Pathological Anatomy, S. Maria delle Croci Hospital, Ausl Romagna, 48121 Ravenna, Italy; silvia.zago@auslromagna.it

**Keywords:** X-ray, skeletal, diagnostic algorithms, projections

## Abstract

Conventional radiography is widely used for postmortem foetal imaging, but its role in diagnosing congenital anomalies is debated. This study aimed to assess the effectiveness of X-rays in detecting skeletal abnormalities and guiding genetic analysis and counselling. This is a retrospective analysis of all post-abortion diagnostic imaging studies conducted at a centre serving a population of over 300,000 inhabitants from 2008 to 2023. The data were analysed using descriptive statistics. X-rays of 81 aborted foetuses (total of 308 projections; mean: 3.8 projections/examination; SD: 1.79) were included. We detected 137 skeletal anomalies. In seven cases (12.7%), skeletal anomalies identified through radiology were missed by prenatal sonography. The autopsy confirmed radiological data in all cases except for two radiological false positives. Additionally, radiology failed to identify a case of syndactyly, which was revealed by anatomopathology. X-ray is crucial for accurately classifying skeletal abnormalities, determining the causes of spontaneous abortion, and guiding the request for genetic counselling. Formal training for both technicians and radiologists, as well as multidisciplinary teamwork, is necessary to perform X-ray examinations on aborted foetuses and interpret the results effectively.

## 1. Introduction

Radiology may assist pathologists’ work in achieving or specifying the diagnosis of congenital anomalies in case of abortion. Diagnostic imaging may also have legal implications or be necessary for genetic counselling, whether abortion is unexpected or induced after an intrauterine diagnosis of congenital anomalies. Moreover, the declining parental consent rates for conventional autopsies [1,2,3] in many developed countries contribute to providing a significant motive to introduce radiology as an unavoidable part of postmortem assessment. 

In this context, some authors are exploring new techniques, such as CT, MRI, ultrasound, and micro-CT, as noninvasive tools for investigating perinatal deaths to completely replace autopsy [4].

However, since micro-CT and high-field MRI (>7 T) are available in a few select centres/research facilities only [5], and due to a lack of accepted international protocols, in everyday clinical practise, the imaging of aborted foetuses can only make use of standard technology, namely CT, MRI, US, and X-ray [5]. 

Among these imaging methods, MRI has the highest concordance with standard autopsy, ranging from 58% [6] to more than 90% [7], with more elevated diagnostic power at higher field strengths (e.g., 3 T) [8]. MRI is particularly used for assessing soft tissue/internal organ detail. However, its availability/access may be limited, and it has a poorer resolution in smaller foetuses (<500 g). 

On the contrary, whole-body postmortem ultrasound (US) is readily available, cheap, and portable, and can be used on any size of foetus [4]. It is used to assess soft tissues and internal organs; diagnostic accuracy rates similar to both 1.5 T [9] and 3 T MRI [10] have been reported, with an estimated overall sensitivity of 73% and specificity of 97% [11].

However, it is an operator-dependent imaging method. It requires a hands-on approach, and imaging quality can be affected by ‘intrauterine retention time’, namely the time between an intrauterine foetal death and delivery, with a relevant fall in its diagnostic power within 48 h [12].

CT is rarely performed in perinatal postmortem imaging because of the lack of soft-tissue contrast [13]. Its indications are limited to bony injuries and trauma considered for skeletal dysplasias or trauma, but radiographs may be better and cheaper [5]. The potential of increasing the detection of some additional abnormalities by using CT remains to be determined clinically [1,5]. Finally, X-rays are easy to perform, are part of routine autopsy services, can be used on foetuses of any size, and help diagnose skeletal abnormalities only. Specifically, postmortem X-rays may be used in the evaluation of skeletal anomalies to confirm any in utero diagnosis as well as specify the diagnosis of congenital abnormalities, which can help in counselling for future pregnancies.

For these reasons, in everyday practise, conventional radiography remains the foetal postmortem imaging service most provided in Europe, Canada, and the USA. It is recommended, among all imaging techniques, to be considered the gold standard of perinatal postmortem imaging [14,15,16]. Conventional radiology allows the diagnosis of bony abnormalities [14], and according to the Royal College of Pathologists guidelines on autopsy practise, a whole-body X-ray for gestational assessment and malformation is recommended in all cases of foetal loss and mandatory for suspected skeletal dysplasia [17]. 

However, there is an ongoing discussion regarding traditional radiology’s exact role in assessing congenital foetal anomalies [1]. Some authors, indeed, claimed that no significant clinical diagnoses would go undetected if plain radiographs were not obtained in cases of apparently structurally normal aborted foetuses and that a routine postmortem plain radiography contributed significantly to the final diagnosis in less than 0.5% of cases [1]. 

A low overall yield of routine and unselected perinatal postmortem skeletal radiography raises doubts about the justification of such an approach, also considering the burden of workloads and significant time and financial cost implications for radiologic units [1].

Moreover, prenatal US currently has high accuracy rates, even for the most complex musculoskeletal findings [18].

Finally, there is a lack of standardised codified procedures:

The lack-of-radioprotection issue leaves open the possibility that there is no clarity on the indications for the request of these investigations by the pathologist, and no clarity on the need to instead perform them routinely, as seems to emerge from a study on the clinical practise of perinatal postmortem radiography [1].

At the same time, although a standard postmortem babygram consists of anteroposterior (AP) and lateral–lateral (LL) images of the total foetus and added images of the indication [19], it is possible that in clinical practise, radiological studies with defined radiological projections are not performed [1] or that in many institutions, non-specific protocols, including complete skeletal surveys, like in nonaccidental trauma, are generically adopted [16].

Given this background, the present study aims to report the actual use of diagnostic imaging over fifteen years at our institution and to explore traditional radiology’s proper role in everyday practise.

The aims of the present work are as follows:

1. Define the role of plain radiology concerning autopsy by calculating its diagnostic power, listing the indications for the pathologist’s radiology requests in aborted foetuses, and defining when radiology should instead be conducted regularly.
2. Specify the role of traditional radiology concerning the data provided by prenatal US. Specifically, we hypothesised a high agreement rate between the first trimester US scan and postmortem radiographs, with a concordance rate similar to that between postmortem US and autopsy (86.4%).
3. Understand whether X-rays may provide any information to address the need for genetic analysis and counselling. We hypothesised the existence of some correlation between radiographic findings and genetic disorders, which could be adopted as specific radiologic targets for the decision to request further genetic analysis.
4. Understand whether a regular practise exists for executing postmortem radiograms in clinical practise. We hypothesised that no radiologic protocol is applied in everyday practise, meaning a mean of more than two standard AP and LL radiograms were performed and that there was a wide range in the number of executed projections.

## 2. Materials and Methods

An Ethical Committee (Comitato Etico della Romagna—CEROM, Meldola-FC, Italy) preliminarily approved this study (n. 2501, 2019), which was conducted according to the Helsinki Declaration of 1975, as revised in 1983. Due to the study’s retrospective nature, the request for parents’ consent was waived.

We preliminarily searched our institution’s Picture Archiving and Communication System (PACS) to identify all post-abortion diagnostic imaging studies from April 2008 to September 2023. For this purpose, we formulated a proper search strategy using the identification strings commonly used to mark these diagnostic exams at our institution. We searched all radiological studies of all imaging techniques performed on aborted foetuses and collected them in subsequent chronological order.

Since our study’s purpose was to assess the role of postmortem radiological imaging, we excluded from our analysis all in vivo studies, such as prenatal US and MRI, whose reports were, however, examined for comparison.

From each radiological report, we extrapolated the radiological diagnosis.

For each included case, two radiologists independently reviewed each examination to confirm the diagnosis, report the number of available radiograms, describe each projection, and report the radiological parameter (i.e., Kv and mA/s) whenever available.

Subsequently, we searched the register of foetal autopsy examinations executed at our institution during the same period. 

We included in our analysis all the cases of abortion where diagnostic imaging had been conducted. 

Exclusion criteria were as follows: the cases of abortion induced for non-therapeutic purposes, the cases of foetal malformations diagnosed after the 28th week, and the cases where the autopsy data were missing, including those in progress.

We subsequently searched for the medical reports in the database of our institution, and we extracted the following:

1. The cause of foetal death.
2. The results of any histological and bacteriological examination.
3. The findings of the ultrasound examination at the 20th week.

We also accessed our institution’s clinical data repository whenever necessary to search for the final diagnosis or diagnostic hypothesis.

Moreover, we asked the genetic institute for the karyotype of the foetuses and the genetic counselling results whenever available. 

Finally, we obtained data on voluntary interruptions in pregnancy and intrauterine deaths per year relative to the province served by our institution over the study’s period.

The data were filled in an Excel datasheet, and statistical analysis was completed using Wizard Pro software (V 1.9.49).

The Excel spreadsheet included the following: 1. Demographic data: 1.1. an anonymising code referring to each mother; 1.2. the mother’s age at the event of the abortion; 1.3. period of amenorrhea (days); 1.4. sex of the foetus; 1.5. date of birth; 1.6. unexpected death (yes/no).

2. Radiological data: 2.1. presence/absence of any radiological anomaly reported in the examined radiological reports; 2.2. number of reported foetal alterations on X-ray; 2.3. radiological anomalies’ localisation and type. 3. Anatomopathological diagnosis. 4. Morphological ultrasound. 5. MRI data: 5.1. Execution of MRI (yes/no); 5.2. MRI results. 6. Genetic tests: 6.1. execution of genetic tests (yes/no); 6.2. genetic test results with any chromosomal anomalies.

Section 2.3 of the Excel spreadsheet was subdivided as follows. a. Craniofacial: microcephaly, turricephaly, non-specific cranial anomalies, occipital dysplasia, micrognathia, cleft palate. b. Vertebral: supernumerary vertebra, transitional vertebra, spina bifida, incomplete vertebral development, sacrum/coccyx anomalies. c. Limbs: upper/lower limb dysplasia, stubby upper/lower limbs, humeral hypoplasia, double ulna, crooked hand, femur hypoplasia, clubfoot, oligodactyly, polydactyly, syndactyly, clinodactyly. d. Other: skeletal dysplasia, costal anomalies, arthrogryposis.

To summarise the characteristics of the dataset, we used descriptive statistics.

To answer the first hypothesis, we used the software Med Calc v. 22.030 to calculate the diagnostic power of radiology. For this purpose, when more than one malformation in the same child/foetus was present, it was counted only once. An estimation of the positive predictive value was calculated by adopting the estimated prevalence of 0.80% reported for musculoskeletal system anomalies in the general population by Doyle et al. [20].

Concerning the second hypothesis, the concordance rate between the first-trimester US scan and postmortem radiographs was calculated.

Regarding the third hypothesis, the presence/absence of any chromosomal anomaly was tested against the presence/absence of each group of anomalies reported in Section 2.3 of the Excel spreadsheet, a. craniofacial, b. vertebral; c. limbs; d. other, and, subsequently, against the presence/absence of each of the 25 specific musculoskeletal anomalies reported in the spreadsheet. Furthermore, we searched for any correlation between the result of a chromosomal anomaly at the genetic tests and the presence of any specific type of skeletal anomaly using the Chi-Square test (critical value @5%: C = 1.96; significance: z > C).

To answer the fourth hypothesis, we calculated the total number of projections included in the X-ray examinations, the mean, the range, and the standard deviation (SD), using Excel Functions.

## 3. Results

The present analysis was conducted at a centre serving an area with over 300,000 inhabitants, where, over the period of 2014–2020, the mean/year of abortions that occurred was 755.6 (median: 736), of which 346.8 were spontaneous abortions and 408.7 were voluntary pregnancy interruptions. In the same area, between 2008 and the first semester of 2019, the number of intrauterine deaths, defined as events classified with code 656.41 or 656.43 according to the International Classification of Diseases, 9th revision—Clinical Modification (ICD 9CM), was 83 (7.21/year; median: 7) [21]. Over 15.4 years, our search found that 87 foetuses underwent a diagnostic imaging examination; we excluded 4 cases that did not have an anatomopathological report, and 2 that only had an in vivo MRI examination. We therefore included 81 aborted foetuses, 65 resulting from therapeutic abortions and 16 from unexpected abortions, that underwent postmortem imaging. 

All the included cases had a total-body X-ray study, with six also having an in vivo MRI examination to define cerebral malformation in foetuses before therapeutic abortions. 

Imaging was obtained in 1.68% of all abortions; all the cases examined with diagnostic imaging subsequently underwent autoptsic examination. 

The mean mother’s age at birth was 33 years old (SD: 6), and the mean number of days of amenorrhea was 146 (SD: 40 days). Among the foetuses, 42 were phenotypically female (52.5%) and 38 were male (47.5%); the data were unknown in 1 case. All therapeutic abortions were performed before the 28th gestation week.

In all cases, the clinical question for the X-ray examination included any skeletal alteration (cranium, maxillofacial bones, spine, ribs, limbs, pelvis, hands, and feet), and a radiology specialist reported the study. 

Our series included 308 projections (mean: 3.8 projections/examination; range 1–13; median: 3; SD: 1.79). The examination included a couple of orthogonal views (AP and LL) in all cases but two. In one case, the examination included an AP projection only, and in one case, the examination consisted of an LL view. 

In 49 cases, the examination included one contralateral whole-body projection; in 33 cases, it required the repetition of one or more whole-body orthogonal views (total number of projections: 63); and in 22 cases, it was completed by unique views (36 projections).

Of the 33 cases requiring repetitions, 18 (total number of projections: 33) involved repetitions of whole-body AP or LL views, specifically obtained to include the entire body of the foetus. In two cases, the foetus was particularly large, while in the remaining cases, repetition was necessary because X-ray cassettes with a field of view that was too small were used. 

Four cases did not specify the cause of repeated AP or LL radiograms.

Moreover, in 17 cases, 26 projections were acquired to eliminate overlaps between the skeletal segments or to obtain orthogonality after placing the limb parallel to the imaging plate to avoid a false appearance of a short bone on the radiography. Specifically, one case required four additional projections to adequately demonstrate a twisted hand, and one required an additional projection to forefront an obligatory foetus position. 

Most of the 36 unique projections were to the lower limbs (*n* = 13; in four cases, they were focused on feet), followed by the upper limbs (*n* = 11; in three cases, they were focused on the hand) and the head (*n* = 7). In four cases, semi-lateral or posteroanterior prone position whole-body projections were acquired. In contrast, a single lateral–lateral projection on the cervical spine was added in one case. The mean kV value was 62.09 (range: 45–90 kV; median: 58 kV; SD: 12.04), and the mean mA/s value was 5.94 (range: 1–16 kV; median: 4 kV; SD: 4.81).

A pathologist with specific experience in neonatology executed all the autopsies. They were conducted according to the standardised institutional protocol. All foetuses were preliminarily photographed, including one shot for each subject: whole body prone, whole body supine, left and right flank, hands, feet, and malformations. Regarding malformation, the pathologist proceeded to a detailed macroscopic description, then weighed and measured all structures. Then, the pathologist performed a macro- and microscopic examination of all organs, bones, osteochondral junctions, and muscles. 

Genetic testing was available in 46 cases (56.7%), resulting in euploidy in 63% of the cases (*n* = 29) and different kinds of alterations in 36.9% (*n* = 17).

Among them were included eleven cases of trisomy, including four cases of mosaicism (two of chromosome 21, one of chromosome 14, and one of chromosome XXY), three cases of trisomy 18, one non-mosaic trisomy 21, one tetrasomy, a case of deletion of chromosome 7 (involving the region q32) associated with microduplication of the terminal region of chromosome 6, a short arm microduplication of chromosome X, a 4p16,3 microdeletion, and one case of triploidy. 

Genetic analysis also revealed one case of abnormality of sexual differentiation in the presence of chromosomal makeup 46 XY. 

X-ray examinations detected 137 skeletal anomalies in 55 foetuses (mean per foetus: 2.49; range 0–7). 

The most common alterations were micrognathia (16%; *n* = 23), followed by clinodactyly (8%; *n* = 11), clubfoot (7.3%; *n* = 10), and incomplete vertebral development (7.3%; *n* = 10).

The distribution of skeletal abnormalities across the anatomic region is reported in Table 1. 

A detailed description of all the reported skeletal abnormalities and their association is reported in Supplementary Materials S1. When considering therapeutic abortions, the number of those showing skeletal abnormalities at X-ray examination was 49 (Supplementary Materials S2).

In the sixteen spontaneous abortions, the radiological study confirmed prenatal ultrasound data of the absence of alterations in nine cases. At the same time, it identified skeletal anomalies in seven patients (Table 2), six of whom the alterations had escaped the morphological ultrasound.

In all the cases of therapeutic abortions (*n* = 65), there were pathological findings in morphological ultrasound performed at the 20th week. 

Among them, radiology did not show abnormalities in 26.1% (*n* = 17). In 73.8% (*n* = 48), X-ray examination confirmed or integrated the ultrasound data, demonstrating the presence of skeletal anomalies. 

**Figure 1:**
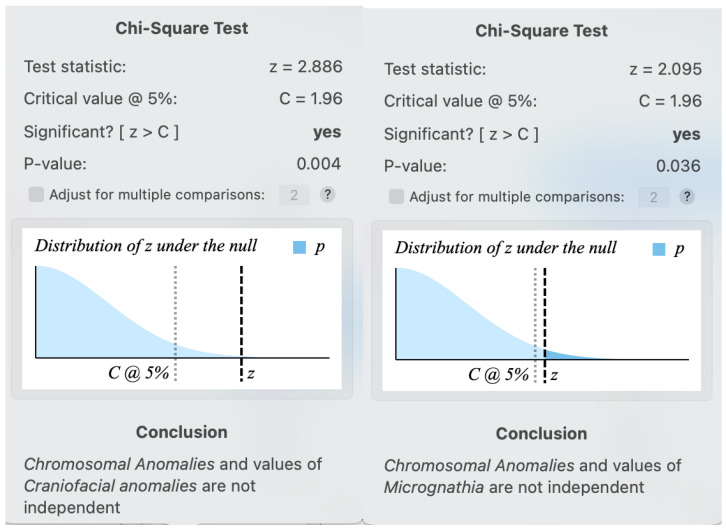
Results of the Chi-Square test, which searches for the correlation between chromosomal anomalies and craniofacial anomalies on X-ray and between chromosomal anomalies and micrognathia.

A positive correlation was found by calculating the Chi-Square test coefficient between the existence of chromosomal anomalies and any craniofacial anomalies (including microcephaly, micrognathia, turricephaly, occipital dysplasia, nonspecific cranial anomalies, and cleft palate; *p*-value: 0.004) and between chromosomal anomalies and micrognathia (*p*-value: 0.036) (Figure 1).

The autopsy confirmed the radiological data in all cases except for two radiological false positives, in which the pathologist did not confirm the radiologic report of micrognathia; in one of these cases, micrognathia was the only musculoskeletal reported anomaly.

On the contrary, in one case of triploidy, radiology missed a diagnosis of syndactyly, which anatomopathological analysis revealed; however, it correctly described other musculoskeletal anomalies.

The diagnostic power of radiology for assessing the existence of any musculoskeletal abnormality (more than one malformation in the same child/foetus counted once only) was 67.5% (95% CI: 56.11–77.55%), and the estimated positive predictive value was 0.54% (95% CI: 0.47–0.63%).

On X-ray, four foetuses showed microcephaly. The condition had already been revealed by morphological ultrasound in three cases, while one case had been further examined in utero with MRI, revealing an associated right micro-ophthalmia.

In the other five cases with in vivo MRI, the reports identified severe cerebral malformations, including vermis hypoplasia, agenesis of the corpus callosum, and type II lissencephaly, so that the correlation with postmortem X-ray reports helped pathologists to diagnose complex syndromes.

## 4. Discussion

Despite the diffusion of modern imaging techniques in clinical practise, plain radiography was the only diagnostic imaging method extensively used at our institution to support pathological diagnosis; these data align with previous reports [22] and current evidence [14,15,16]. Indeed, over the considered period, at our centre, imaging on aborted foetuses was performed on a mean of 5.8 cases/year, reflecting a use limited to a small subset of aborted foetuses. These data fall within the range of 1 to 20 cases/year, perinatal death included, reported by 91% of the centres [16].

A recent survey in North America confirms that 78% of the institutions use radiographs to assess skeletal disorders in aborted foetuses thanks to their availability, rapidity of execution, and reliability. In contrast, postmortem CT and MRI use is still limited due to a lack of funding and formal training, which are crucial for the radiologist to read these examinations adequately [16].

This picture seems to have remained unchanged over time since a similar survey conducted more than ten years ago by the European Society of Paediatric Radiology (ESPR) reported that radiographs were used in 81% of centres, with CT and MRI being adopted in 51% and 38% of centres, respectively [23].

In this context, it is fair to update the state of the art and report data about the actual use of traditional radiology in the study of aborted foetuses, also considering the paucity of the data in the literature.

Indeed, most of the recent literature focuses on using imaging to replace anatomopathological examination, pointing in the direction of a virtual autopsy [4,5,24] rather than on the indications and protocols of traditional radiology.

On the other hand, apart from many interesting case reports, older series are mainly focused on the use of radiographs in specific subsets of cases, like unexpected and unexplained deaths [25], or on particular conditions, like aneuploidies, Down’s syndrome, or skeletal dysplasia [26,27,28].

Furthermore, many studies included foetal and paediatric age groups, ranging from neonates to adolescents [14,15,16,23]. 

### 4.1. Information That Traditional Radiology Can Provide to Support the Pathologist’s Work

Among the information that traditional radiology can provide to support the pathologist’s work in our series, we found the examination and interpretation of morphological anomalies evident on external examination, providing critical information before autopsy distorts the anatomy.

**Figure 2:**
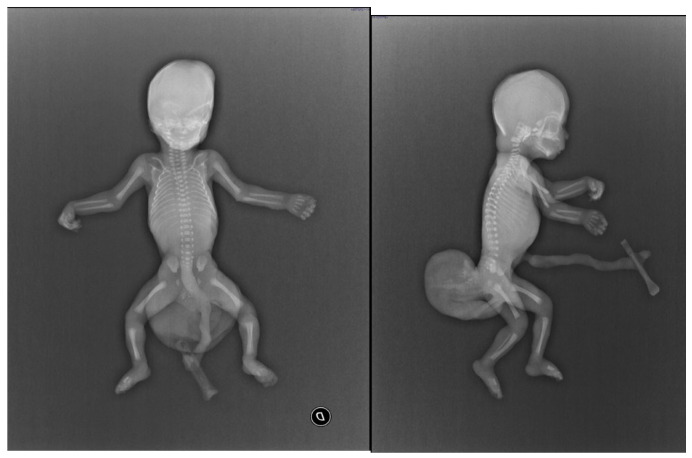
Immature sacrococcygeal teratoma. X-ray examination revealed a soft tissue mass with extra-foetal extension.

For example, X-ray was used to evaluate small thoraxes or dysmorphisms (Figure 2) and guided selective bone resection during autopsy, as Srigg and Whitby previously reported [22]. 

Since specific sections of the extremities are affected in many foetal disorders [29], plain X-ray was crucial for carefully classifying skeletal abnormalities, differential diagnoses, and complex diagnoses such as VATER syndrome, osteogenesis imperfecta, or campomelic dysplasia. For example, in our series, the radiography, by adding key aspects complementary to the anatomopathological data, contributed to classifying a case of type 1 thanatophoric dysplasia and a case of type 2 thanatophoric dysplasia, in which inaccurate classification could lead to incorrect indications of the risk of recurrence in a future pregnancy [22]. In another case, radiographic imaging revealed the association between micrognathia and foetal pneumonia, a crucial result since congenital infections could explain specific skeletal structural abnormalities [22]. 

In addition, radiography proved to be a critical tool for examining vertebral anomalies, which may be essential for addressing or completing the diagnosis. In our series, vertebral abnormalities in aborted foetuses represented 18.2% (*n* = 25) of all skeletal anomalies. All cases were associated with chromosomic anomalies, other skeletal anomalies, or severe malformations affecting the central nervous system, the respiratory system, or the genitourinary apparatus. Scientific literature data confirmed that a vertebral defect can occur alone or with coexisting anomalies and may be part of syndromic findings or Down syndrome [30,31]. For example, in a study on the radiographs of 374 deceased foetuses and infants, an abnormal vertebral pattern was associated with congenital abnormalities in 73.3% [32]. Similarly, a radiographic study on second-trimester foetuses reported costovertebral abnormalities in 75.2% of aneuploid foetuses, trisomy 18 in particular [26]. A higher prevalence of supernumerary cervical vertebrae was previously found in stillborn and deceased infants than in their controls (54.8% vs. 1.1%) [33], supporting the theory of strong selection against changes in the number of cervical vertebrae in humans [34]. 

In this context, the regular use of traditional radiology may help shed light on the precise association of vertebral abnormalities with other skeletal or soft tissue abnormalities, which is still controversial [35]. 

In conclusion, the pathologist can request postmortem radiology with the following indications.

1. Interpretation of morphological anomalies evident on external examination;
2. Providing critical information before autopsy distorts the anatomy;
3. Guiding dissection procedures and selective bone resection during autopsy;
4. Classifying skeletal abnormalities, identifying syndromes, and narrowing down the differential diagnosis of genetic disorders;
5. Supporting the discovery of possible associations between skeletal abnormalities and internal-organ pathological conditions;
6. Examining vertebral anomalies.

**Figure 3:**
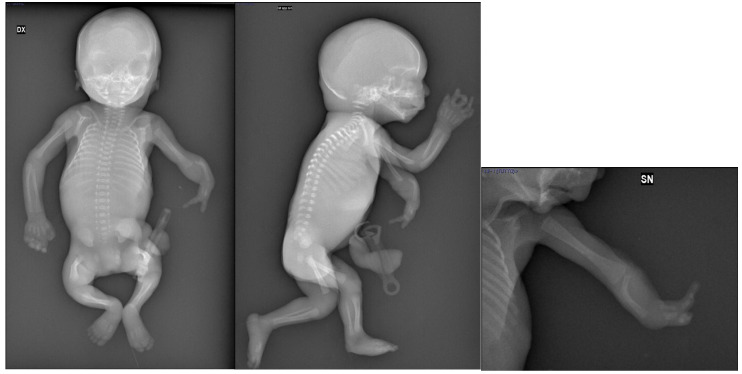
Skeletal dysplasia: whole-body anteroposterior and latero–lateral view and adjunctive on the upper limb showing ulna recurvation. DX: Right; SN: Left.

Regular postmortem radiography should be reserved for specific clinical settings identified before autopsy examination, such as skeletal dysplasia (Figure 3) and complex genetic syndromes.

### 4.2. Role of Traditional Radiology Concerning Prenatal US

Among the indications of X-rays in our series was the study of the causes of intrauterine foetal death. According to a previous study aiming to assess the value of foetal skeletal radiographs in determining the aetiology of foetal death, skeletal abnormalities were detected in 404 foetuses (33.9%), of which minor abnormalities were observed in 271 (22.7%), and severe abnormalities in 173 (14.5%) [25]. Our data show that the radiological definition of skeletal abnormalities is essential in defining the cause of death, often leading to a suspect of a genetically determined syndrome or multisystem disorder. We therefore confirm the role of imaging, X-ray included, in guiding the request for genetic counselling and the management of a future pregnancy [36,37].

The concordance rate between the first-trimester US scan and postmortem radiographs was 70.37% (*n* = 57). On the one hand, radiologic findings might confirm antenatal imaging and complete a diagnosis by providing additional elements. More in detail, thanks to X-ray examination, skeletal abnormalities were confirmed in 73.8% (*n* = 48) of therapeutic abortions and revealed in 43.7% (*n* = 7) of spontaneous abortions. On the other hand, we documented that in 12.7% (*n* = 7) of the cases, the radiological abnormalities detected by postmortem radiology were missed by prenatal ultrasound examination, a percentage which is much higher than the previously reported data of 0.75% (9/1193) [25]. This result is unexpected, considering that the prenatal morphological examination has dramatically improved in the modern day thanks to technological improvement and specific training courses. However, fetal movements often reduce the detection rate of musculoskeletal disorders, especially in low-risk pregnancies, and this underlines the role of postmortem X-ray examination [38].

### 4.3. Can X-rays Address the Need for Genetic Analysis and Counselling?

In our series, limb defects (58.7; *n* = 41) and maxillofacial defects (30%; *n* = 21) were the most common alterations. With inclusion criteria similar to those adopted in our study, Bourlière-Najean described a series where the most common anomalies were abnormal rib numbers (13.4%; *n* = 160) and spinal abnormalities (5.3%, *n* = 63) [25]. Since a different diagnostic power of X-ray examination can be excluded despite eventual different execution conditions, and because our data were confirmed by anatomopathological examination, such different results probably depend on a different incidence of foetal skeletal conditions, which may vary in different populations, geographic locations, and at different times, with genetic factors playing a crucial role. For this reason, we investigated any association between skeletal abnormalities and the results of genetic analysis. 

In the scientific literature, developmental disorders of certain parts of the extremities (dysmelia) occur in 3 to 4 of 1000 pregnancies [29] and are associated with chromosomal anomalies. Bots et al. explained that aneuploidies cause gene–dosage imbalances, presumably resulting in limb fluctuating asymmetry in deceased human foetuses and infants. As a result, limb asymmetry was 1.5 times higher for aneuploids like trisomy 13, trisomy 18, monosomy X, and triploidy than for trisomy 21 patients, and both reference groups had higher life expectancies [39]. Despite this background, we did not find limb malformations related to genetic or chromosomal aberrations.

Instead, we found a positive correlation between maxillofacial anomalies and genetic abnormalities. The association between absent nasal bone and common autosomal trisomies is well known in the literature; indeed, ultrasound screening for nasal bone anatomy is part of routine clinical practise [40,41]. It was also already known that the maxilla is significantly shorter in foetuses with trisomy 21, 18, and 13, as well as with triploidy [42], and micrognathia can be associated with genetic disorders and foetal syndromes, aiding the identification of a recognisable pattern of malformation when associated with cardiac or lymphatic anomalies [43]. On the contrary, cleft palate is known to be of multifactorial origin, and its occurrence may vary in different populations. However, most factors in its aetiology remain to be exhaustively investigated [44]. 

Our finding of a positive correlation between radiologic evidence of maxillofacial abnormalities and genetic anomalies suggests that such anomalies could be adopted as specific radiologic targets for the decision to request further genetic analysis. 

Nevertheless, the possibility of a false positive must be considered when assessing these conditions, since we reported the case of a radiological false positive.

In conclusion, further research may confirm the hypothesis that maxillofacial radiological abnormalities are used as a knot in a decision-making process that leads to genetic counselling.

### 4.4. What Are the Pitfalls in Aborted Foetus X-ray Examinations?

**Figure 4:**
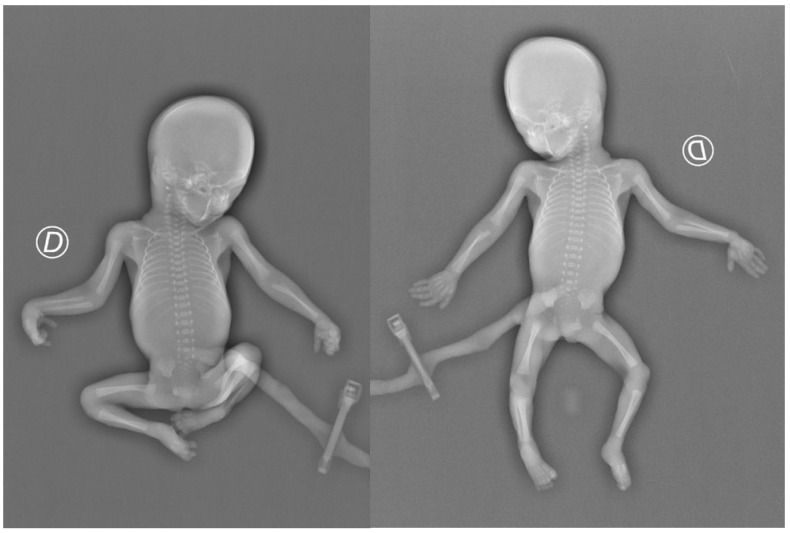
Repetition of X-ray after extension of the limbs to limit artefacts.

While almost all centres include an AP whole-body babygram in their protocol for X-ray studies in foetal deaths, some do not perform a lateral babygram view. The number and exact nature of radiograms obtained vary, and many centres include oblique chest views [23]. The lack of widely accepted protocols reflects the high number of projections reported in our study. While in our centre, two orthogonal views are generally required for a standard X-ray study, we also found that multiple images were necessary to finalise a diagnosis in specific cases, significantly limiting projective artefacts. In particular, when a foetus presents a limb flexed in a forced position, it may be necessary to obtain further images after placing the limb parallel to the imaging plate to avoid the false appearance of a short bone on radiography (Figure 4).

Proper exposure may significantly vary between an early spontaneous abortion and a full-sized foetus. Since a short exposure time, usually adopted in vivo to reduce motion artefacts, is not required, the exposure may be achieved by adjusting exposure times rather than altering kilo-voltage (kV).

Low-kV X-rays are commonly combined with dedicated fine-detail phosphor plate detectors to maximise contrast and spatial resolution [22]. Based on these observations, we speculated that technicians should be specifically trained to perform X-ray examinations of aborted foetuses correctly. They should be able to set radiographic parameters, capture adjunctive projections whenever necessary, and correctly position the foetus on the imaging plate to optimise the imaging results. 

Based on the results of the present study, we recommend searching for orthogonality, eliminating every space between each studied anatomic part and the cassette, eliminating overlaps, and using cassettes of a size adequate to the foetuses’ dimensions.

Further studies should focus on developing a standardised protocol and reaching a consensus on the exact role of X-rays in foetal postmortem diagnostic imaging concerning cross-sectional imaging and US.

### 4.5. What Organisational Measures Can Help the Use of X-rays in Postmortem Foetal Diagnosis?

We observed that some of the reported diagnoses were not obvious, with a missed diagnosis and two false positives in our series requiring a high suspicion index. Among the causes is the high frequency of skeletal disorders in the foetuses, with an incidence of approximately 5:1000 pregnancies and more than 450 skeletal malformations associated with the syndromes or genetic anomalies described [29]. 

Therefore, we argue that reporting foetal traditional X-ray examinations requires specific experience in foetal pathological conditions. Our finding of two cases of false positivity further underlines the need for technicians and radiologists to receive formal training in foetal postmortem diagnostic imaging [22]. Our results also support the claim recently formulated by Harty et al. [16] that collaborative working relationships within a multidisciplinary team are crucial for the postmortem imaging of aborted foetuses. 

For example, knowledge of any abnormality diagnosed through the antenatal US may help the radiologist interpret any suspicion of bony abnormalities on the X-ray correctly. Knowing the foetus’s gestation age is critical when assessing bone length. At the same time, the crown–rump length (CRL) availability recorded during the first-trimester US scan may be a critical reference point to confirm the radiologic data.

Furthermore, the radiologist may find it difficult or impossible to identify soft tissue abnormalities, so the pathologist must specify on the request card any relevant result of the initial inspection (e.g., fused digit or limb deformation). 

For the reasons above, specific clinical audits, inter-departmental cooperation, a PACS, and a clinic data repository are critical. They should be implemented wherever necessary to improve good practise and service quality. Moreover, centralised information regarding foetal postmortem diagnostic imaging service provision is advisable [16].

### 4.6. Limitations

The main limitation of the present study is that it is restricted to the series of a single centre, which does not allow us to draw generalisable conclusions.

No postmortem CT cases were included in our study, which confirms the evidence of a survey from the European Society of Paediatric Radiology (ESPR) that CT in foetal postmortem assessment is still scarcely used, mainly due to increased reporting times, a lack of reporting expertise, and limited scanner availability [15].

At the same time, however, we reported up to 13 projections for a single foetus. CT could have been more efficient and precise in describing skeletal malformations in cases with twisted, distorted, complex bony structures challenging to examine in detail through plain radiography, which is a perfect imaging technique for preliminary assessment.

Therefore, future studies should address diagnostic algorithms by clarifying the relative role of each diagnostic imaging method, similar to what has already been carried out in other branches of medicine.

In our centre, in vivo MRI was never used to assess musculoskeletal abnormalities or integrate ultrasound diagnoses of anomalies other than those affecting the central nervous system.

As previously reported in the scientific literature [45], using this imaging technique to examine the body’s anomalies like oligohydramnios and diaphragmatic hernias may be time-consuming and technically demanding. It requires dedicated, specifically trained radiologists and tight cooperation with pathologists and foetal medicine specialists. 

Furthermore, our series used only traditional radiology, and more modern techniques such as US, CT, or postmortem MRI were not used.

However, our analysis was aimed at photographing the actual use of diagnostic imaging in clinical practise, and the fact that other methods were not used is a result that we thought was fair to discuss rather than an actual limitation of this study.

Furthermore, our analysis focused on radiography to better define its diagnostic role, purposes, and limitations concerning pathological anatomy, genetic data, and in vivo US, considering it a tool to guide proper access to second-level imaging methods that are more expensive and complex and less available.

The number of cases examined was limited in absolute terms, but postmortem diagnostic imaging was used only in limited ways [16]. Therefore, this may be considered a fairly extensive series of cases covering fifteen years. 

Finally, the study’s retrospective nature has probably introduced no significant biases since the aim was to examine the method’s actual use, a purpose for which the PACS archive and clinical databases are the ideal data sources.

## 5. Conclusions

Plain radiography was the only postmortem diagnostic imaging method extensively used at our institution to support pathological diagnosis in aborted foetuses.

An X-ray allows the interpretation of morphological anomalies evident on external examination, providing critical information before autopsy distorts the anatomy. It is crucial to classify skeletal abnormalities, including vertebral ones, carefully, address differential diagnoses, and allow complex diagnoses.

Moreover, in our series, the radiological definition of skeletal abnormalities proved to be essential in defining the cause of intrauterine foetal death and guiding the decision on counselling for future pregnancies.

Our finding of a positive correlation between maxillofacial radiological abnormalities and genetic anomalies could be explained by the fact that maxillofacial anomalies are the phenotypic result of multigenetic interactions and, therefore, could reveal an index of specific genetic anomalies. Such malformations could be adopted as a specific target for the decision to request further genetic analysis. However, further research is required to confirm the reported correlation, clarify which precise genetic anomalies are associated with the described morphological anomalies, and define which genetically determined syndromes they are involved in.

Based on our observations, both technicians and radiologists should receive formal training to perform X-ray examinations on aborted foetuses, and a collaborative working relationship within a multidisciplinary team is vital for the postmortem imaging of aborted foetuses.

Although the present study was restricted to a single centre, it covered 15 years, and our data could support future research mainly focused on the formulation of diagnostic algorithms by clarifying the relative role of each diagnostic imaging method, including postmortem CT and MRI.

## Figures and Tables

**Table 1:** Breakdown of radiologically detected skeletal abnormalities per anatomic region.

Anatomic Region	Number	Radiological Finding
Maxillofacial	25	Micrognathia (23), cleft palate (2).
Limbs	52	Clinodactyly (11), clubfoot (10), short limbs (5), clubhand (3), syndactyly (3), limb dysplasia (6), femoral hypoplasia (3), double ulna (2), polydactyly (2), oligodactyly (5), humeral hypoplasia (2).
Vertebrae	25	Transitional vertebrae (6), incomplete vertebral development (10), sacrococcygeal anomalies (6), supernumerary vertebra (2), spina bifida (1).
Cranial	21	Nonspecific cranial anomalies (9), turricephaly (4), microcephaly (4), occipital dysplasia (4).
Other	14	Arthrogryposis (5), nonspecific skeletal dysplasia (3), rib anomalies (6).

**Table 2:** Autopsic and morphological ultrasound findings in unexpected abortions with radiological anomalies.

Case n.	Number of Rx Anomalies	Type of Rx Anomalies	Pathological Diagnosis	Genetic Test Results
1	1	Micrognathia	None	Not executed
2	1	Turricephaly	Atretic brain-myelocele and cerebellar worm agenesis	Microduplicazione braccio corto cr x
3	3	Clubfoot, stocky limbs, clinodactyly	Intrauterine foetal death caused by a hypoxic state due to thrombosis of the vessels of the funiculus	Not executed
4	2	Aspecific cranial anomalies, Sacrococcygeal anomalies	Intrauterine foetal death and polyhydramnios in VACTERL/VATER	Not executed
5	2	Oligodactyly, transitional vertebrae	Chorioamnionitis with placental abruption	Not executed
6	1	Micrognathia *	Wharton gelatin reduction, Intrauterine foetal death in Smith–Magenis Syndrome	Normal
7	4	Turricephaly, incomplete vertebral development, micrognathia, transitional vertebrae	Onphanocele + diaphragmatic eventration (blastogenesis defect)	Normal

* These data were not confirmed by anatomopathology.

## Data Availability

The data supporting the reported results are included in this paper’s tables. The native data can be released by the authors upon motivated request.

## Supplementary Materials

The following supporting information can be downloaded at https://www.mdpi.com/article/10.3390/jimaging10100242/s1, Supplementary Materials S1: Description of all the reported skeletal abnormalities and their association. Supplementary Materials S2: Therapeutic abortions, showing skeletal abnormalities at X-ray examination.
