# Supplementary material for: The Role of Plain Radiography in Assessing Aborted Foetal Musculoskeletal Anomalies in Everyday Practice
Source: J Imaging. 2024 Sep 27;10(10):242. doi: 10.3390/jimaging10100242 (PMC11508908; doi:10.3390/jimaging10100242)
Supplement: Supplementary file 1 [file jimaging-10-00242-s001.zip › Supplementary Materials S1.pdf]

Supplementary Materials S1: description of all the reported skeletal abnormalities and their association.

**Anatomopathological +/- genetic diagnosis**	**Radiologic findings**																									
	Cranial abnormalities				Maxillofacial abnormalities		Vertebral abnormalities					Costal anomalies	Limb abnormalities												Skeletal dysplasia	Number of skeletal alterations
	Turricephaly	Nonspecific cranial anomalies	Occipital dysplasia	Microcephaly	Micrognathia	Palatoschisis	Transitional vertebrae	Supernumerary vertebra	Incomplete vertebral development	Spina bifida	Sacrococcygeal anomalies		Arthrogryposis	Short limbs	Limbs dysplasia	Syndactyly	Clinodactyly	Humeral hypoplasia	Polydactyly	Oligodactyly	Double ulna	Clubhand	Femoral hypoplasia	Clubfoot		
Mosaic trisomy 14							■										■							■		3
Chronic villitis/ Intervillitis																										0
Encephalocele in Joubert **Sdr.**	■		■																■							3
Spontaneous abortion					■																					1
AVID Sdr.													■			■	■									3
COFS Sdr.		■	■	■	■		■		■				■													7
TTTS Sdr.																										0
TTTS Sdr.																										0
bilateral retinoblastoma and CAT																										0
Down Sdr. (therapeutic abortion)																										0
Hypoplasia and dysmorphism of the cerebellar vermis																										0

Corpus callosum agenesis, intestinal malrotation, adrenal hyperplasia, bilateral renal abnormalities																	■									1
Polymarformative Sdr. (limb hypoplasia, cleft lip, right lung with four lobes, penis-scrotum fusion)		■			■						■			■									■			5
FADS					■								■													2
ASD, left dolichoureter and pyelectasis (HOS)																										0
Atresic brain-myelocele and cerebellar vermis agenesis	■																									1
Placental insufficiency and fetal pulmonary hypoplasia																	■					■				2
OI type 2 (therapeutic abortion)		■										■			■		■									4
DWM + VSD																										0
Corpus callosum agenesis					■																					1
Klinefelter syndrome					■																					1
Wolf-Hirschhorn syndrome		■			■																			■		3
Hydrops Fetalis Secondary to Parvovirus B19 Infections																										0
Fetus weighing less than gestation period (16th week) and intrauterine pneumonia					■																					1
Down syndome			■																							1
Corpus callosum agenesis (therapeutic abortion)					■											■										2
Sex developmental disorder with visceral polymalformations					■																					1
Thanatophore dysplasia type 1												■		■												2
Klinefelter syndrome	■																									1
Silver-Russel syndrome				■																	■					2
PProm in anhydramnios and bilateral clubfoot																								■		1
Interhemispheric hamartoma and agenesis of the corpus callosum																										0

Edwards syndrome		■																								1
Skeletal dysplasia of the lower limbs (therapeutic abortion)						■								■			■								■	4
Intrauterine death caused by hypoxic state from thrombosis of the vessels of the funiculus														■			■							■		3
Mirror hand syndrome (Type A: Sandrow syndrome = ulna duplication)																			■		■	■				3
Impaired head / femur development																										0
Intrauterine death in polydramnios and VATER/VACTER syndrome		■									■															2
Polymalformation from amniotic bridle					■											■	■									3
Craniofrontal nasal dysplasia		■			■									■										■		4
Sacrococcygeal teratoma with generalized hydrops due to congestive heart failure																										0
Therapeutic abortion for chromosomal abnormality					■		■																			2
Placental insufficiency																										0
Facial femur syndrome						■																	■	■		3
Thanatophore dysplasia type 2 (cloverleaf skull and thoracic hypoplasia)		■									■	■			■											4
Down syndrome																										0
Univentricular heart, ASD, VSD, aortic arch hypoplasia																										0
Edwards syndrome					■																					1
Bladder exstrophy-epispadias complex																										0
Sepsis following PProm																										0
Non-immune hydrops fetalis																										0
Upper limb anomaly																		■		■						2
Arnold Chiari 2, meningocele										■														■		2

Transposition of the great vessels																										0
Absolute shortness of the funiculus, PProm, chorioamnionite																										0
Chorioamnionitis with placental abruption							■													■						2
Fusinisitis, after cerclage																										0
Retroplacental hematoma in fetus with translocation t(2;7)(q36;q22).																										0
Wharton gelatin reduction in Smith-Magenis syndrome					■																					1
Dandy-Walker syndrome																	■									1
Right microphthalmia / microcephaly				■				■																		2
Immature sacrococcygeus teratoma																										0
Meckel-Gruber's syndrome (ciliopathy), hydropic fetus, pulmonary hypoplasia				■					■																	2
Omphanocele + diaphragmatic eventration (blastogenesis defect)	■				■		■		■																	4
Corpus callosum agenesis, renal anomalies					■		■										■							■		4
Chorioamnionitis and intervillitis in fetus with vermis hypoplasia, gallbladder atresia, scoliosis									■																	1
Overriding aorta, VSD, midgut malrotation																										0
Cardiac malformation, hydrocephalus			■		■												■					■				4
**Tubulinopathy (agenesis of the corpus callosum** and cortical abnormalities)																										0
Single umbilical artery, esophageal atresia, right ventral hypoplasia, vermis hypoplasia (VACTERL)					■				■																	2
Fuhrmann Sdr.															■					■					■	3

Esophageal, radius and thumb atresia												■			■					■						3
Single umbilical artery, complex VACTERL-type and MURCS-type alterations								■	■			■			■					■						5
CRS, cardiac alterations, renal and bladder agenesis, anal atresia					■				■		■				■			■					■		■	7
Dandy Walker + vertebral and facial alterations					■				■		■															3
Flexed wrists and club feet, fewer ribs, urethrovaginal fistula, imperforate anus												■	■											■		3
Fetus of increased weight for the gestational age (unexpected abortion)																										0
Triploidy; facial dysmorphism, syndactyly, pulmonary hypoplasia, corpus callosum agenesis, cerebellar hypoplasia, unilateral renal cystic dysplasia					■																					1
CRS									■		■															2
VSD, abdominal cysts, imperforate anus, hypoplastic cerebellum, clubfoot, flexed lower limbs													■											■		2
Fetal facial lymphangioma (therapeutic abortion)		■			■				■																	3
Total	4	9	4	4	23	2	6	2	10	1	6	6	5	5	6	3	11	2	2	5	2	3	3	10	3	137

ASD: Atrial Septal Defect; AVID: Asymmetric Ventriculomegaly, Interhemispheric cyst, and Dysgenesis of the corpus callosum; CAT: common arterial trunk; COFS: Cerebro-Oculo-Facio-Skeletal; CRS: Caudal Regression Sequence; DWM: Dandy Waljer Malformation; FADS: Fetal Akynesia Deformation Sequence; HOS: Holt-Oram Syndrome; OI: Ostegenesis imperfecta; PProm: Preterm Premature Rupture of Membranes; TTTS: Twin-to-Twin Transfusion Syndrome; VSD: Ventricular Septal Defect
