# Supplementary material for: The Role of Plain Radiography in Assessing Aborted Foetal Musculoskeletal Anomalies in Everyday Practice
Source: J Imaging. 2024 Sep 27;10(10):242. doi: 10.3390/jimaging10100242 (PMC11508908; doi:10.3390/jimaging10100242)
Supplement: Supplementary file 1 [file jimaging-10-00242-s001.zip › Supplementary Materials S2.pdf]

Supplementary Materials S2: Therapeutic abortions, showing skeletal abnormalities at X-ray examination.

Case n.	Number of Rx anomalies	Type of Rx anomalies	Pathological diagnosis	Genetic test results
1	3	Clinodactyly, clubfoot, transitional vertebrae	Mosaic trisomy 14	Mosaic 7 xx + 14
2	3	Polydactyly, Turricephaly, Occipital dysplasia	Joubert syndrome	Normal
3	3	Arthrogryposis<br>Clinodactyly, Syndactyly	AVID[1]	Normal
4	7	Microcephaly, transitional vertebrae micrognathia, occipital dysplasia, aspecific cranial anomalies, incomplete vertebral development, arthrogryposis	COFS [2] syndrome	Normal
5	1	Clinodactyly	Agenesis of the corpus callosum,<br>intestinal malrotation, bilateral adrenal hyperplasia	Normal
6	5	Sacrococcygeal anomalies, femoral hypoplasia, nonspecific cranial anomalies, short limbs, micrognathia	Polymalformative syndrome (limb hypoplasia, cleft lip, right lung with four lobes, webbed penis)	Normal
7	2	Arthrogryposis, micrognathia	FADS[3]	Normal
8	2	Radial Aplasia,<br>clinodactyly	Placental insufficiency,<br>pulmonary hypoplasia	Unknown
9	4	Clinodactyly, costal anomalies, limb dysplasia, aspecific cranial anomalies	Osteogenesis imperfecta type 2	Unknown
10	1	Micrognathia	Agenesis of the corpus callosum	Unknown
11	1	Micrognathia	Klinefelter syndrome	47xxy
12	3	Aspecific cranial anomalies, micrognathia, clubfoot	Wolf-hirschorn syndrome	4p16,3 microdeletion
13	1	Micrognathia	Low fetal weight (at 15-16 weeks) with intrauterine pneumonia	Unknown
14	1	Occipital Dysplasia	Down syndrome	Mosaic 47 xx+21
15	2	Syndactyly, Micrognathia	Agenesis of the corpus callosum	Unknown
16	1	Micrognathia	Disorder of sexual development (DSD) with visceral polymalformations	46,xy DSD

17	2	Costal anomalies, short limbs	Thanatophoric dysplasia type 1	Normal
18	1	Turricephaly	Klinefelter syndrome	Mosaic 47 xxy
19	2	Microcephaly, double ulna	Silver-Russel syndrome	Normal
20	1	Clubfoot	Polyhydramnios,<br>bilateral clubfoot	Unknown
21	1	Nonspecific cranial anomalies	Edwards Syndrome	47 xy +18
22	4	Clinodactyly, skeletal dysplasia, cleft palate, short limbs	Lower limb dysplasia	Unknown
23	3	Radius aplasia, double ulna, polydactyly	Mirror hand syndrome (double ulna)	Normal
24	3	Clinodactyly, syndactyly, micrognathia	Amniotic band syndrome	Unknown
25	4	Nonspecific cranial anomalies, micrognathia, short limbs, clubfoot	Craniofrontonasal dysplasia	Normal
26	2	Transitional vertebrae, micrognathia	Chromosomal anomaly	48xx
27	3	Femoral hypoplasia, clubfoot, cleft palate	Femoral facial syndrome	Normal
28	4	Rib anomalies, limb dysplasia, sacrococcygeal anomalies, nonspecific cranial anomalies	Thanatophoric dysplasia type 2	Unknown
29	1	Micrognathia	Edwards Syndrome	47 xy +18
30	2	Humeral hypoplasia, oligodactyly	Upper limb malformations	Normal
31	2	Spina bifida, clubfoot	Arnold Chiari malformation type 2, meningocele	Normal
32	1	Clinodactyly	Dandy Walker Syndrome	Normal
33	2	Supernumerary vertebrae, microcephaly	Right microphthalmia/microcephaly	46 xx with 7q32 deletion and microduplication of the terminal region of chromosome 6
34	2	Microcephaly, incomplete vertebral development	Meckel-Gruber syndrome<br>(ciliopathy)	Normal
35	4	Turricephaly, micrognathia, incomplete vertebral development, transitional vertebrae	Omphalocele; Diaphragm Eventration (blastogenesis defect)	Normal
36	4	Micrognathia,<br>clinodactyly, transitional vertebrae, clubfoot	Agenesis of the corpus callosum, renal malformations	Normal
37	1	Incomplete vertebral development	cerebellar vermis hypoplasia, gallbladder atresia, scoliosis	Unknown
38	4	Occipital dysplasia, micrognathia,<br>radius aplasia, clinodactyly	Edwards Syndrome,<br>Heart defects, hydrocephalus	47 xy + 18

39	2	Incomplete vertebral development, micrognathia,	VACTERL single umbilical artery, esophageal atresia, right ventricular hypoplasia, vermian hypoplasia.	Normal
40	3	Oligodattilia,<br>displasia art sup/inf<br>displasia scheletrica	Sindrome di Fuhrmann	Not executed
41	3	Anomalie costali<br>displasia art sup/inf<br>oligodattilia	esophageal atresia, radius and left hand radius atresia	Not executed
42	5	vertebra sopranumeraria<br>oligodattilia<br>incompleto sviluppo vertebrale<br>displasia art sup/inf<br>anomalie costali	single umbilical artery+<br>VACTERL + MURCS	Not executed
43	7	ipoplasia omero<br>micrognazia<br>incompleto sviluppo vertebrale<br>ipoplasia femore<br>anomalie sacro/coccige<br>displasia art sup/inf<br>displasia scheletrica	Caudal Regression Syndrome (CRS)+ cardiac alterations, renal and bladder agenesis, anal atresia, limb alteration	Not executed
44	3	anomalie sacro/coccige<br>incompleto sviluppo vertebrale<br>micrognazia	Dandy Walker Sdr + vertebral and facial alterations	Not executed
45	3	piede torto<br>artrogriposi<br>anomalie costali	flexed wrists and club feet, reduced number of ribs, urethrovaginal fistula, imperforate anus	46xx
46	1	micrognazia	facial dysmorphism, syndactyly, pulmonary hypoplasia, corpus callosum agenesis, cerebellar hypoplasia, unilateral renal cystic dysplasia.	triploidia
47	2	incompleto sviluppo vertebrale<br>anomalie sacro/coccige	Caudal Regression Syndrome (CRS)	46xx
48	2	piede torto<br>artrogriposi	Ventricular septal defect, abdominal cysts, imperforate anus, hypoplastic cerebellum, clubfoot, flexed lower limbs	Not executed
49	3	Micrognazia*	Fetal lymphangioma of the face	Not executed

		anomalie craniche aspecifiche<br>incompleto sviluppo vertebrale		

[1]AVID: Asymmetric ventriculomegaly, interhemispheric cyst, and corpus callosum dysgenesis.
[2]COFS: (Pena-Shokeir, type 2); Cerebro-oculo-facio-skeletal.
[3] FADS: **(**Pena-Shokeir, type 1); Fetal akinesia deformation sequence (FADS).
*This data was not confirmed by anatomopathology
